# Pathological complete response to neoadjuvant chemotherapy in triple negative breast cancer – single hospital experience

**DOI:** 10.1186/s13053-023-00249-1

**Published:** 2023-03-16

**Authors:** Elina Sivina, Lubova Blumberga, Gunta Purkalne, Arvids Irmejs

**Affiliations:** 1 Institute of Oncology, Tumour Clinical Research Department, Riga Stradins University, Riga, Latvia; 2 Clinic of Oncology, Pauls Stradins Clinical University Hospital, Riga, Latvia; 3 Department of Internal Diseases, Riga Stradins University, Riga, Latvia; 4 Breast Unit, Department of Surgery, Pauls Stradins Clinical University Hospital, Riga, Latvia; 5 Department of Surgery, Riga Stradins University, Riga, Latvia

**Keywords:** Triple-negative cancer, Neoadjuvant chemotherapy, Complete pathological response, *BRCA*

## Abstract

**Background:**

Triple-negative breast cancer is a heterogeneous molecular subtype of BC. Pathological complete response (pCR) is an important surrogate marker for recurrence-free and overall survival.

**Aim of study:**

The aim of this study was to evaluate clinical and pathological factors that are associated with complete pathological response status in triple-negative breast cancer patients receiving neoadjuvant chemotherapy.

**Materials and methods:**

Eighty triple-negative breast cancer patients who underwent neoadjuvant chemotherapy followed by surgery at Pauls Stradins Clinical University Hospital between January 2018 and January 2020 were retrospectively analysed. Twenty-six patients (32.5%) were *BRCA1/2* pathogenic variant carriers.

**Results:**

A total of 32.5% (*n* = 26) of patients in all study groups and 57.7% (*n* = 15) of patients with *BRCA1/2* pathogenic variants achieved pCR. Forty-seven patients received platinum-based neoadjuvant chemotherapy, and 19 patients (40.4%) achieved complete pathological response. Patients in the pCR group presented with significantly higher Ki-67 scores (*p* = 0.007), *BRCA1/2* pathogenic variants (*p* = 0.001) and younger age (*p* = 0.02) than those in the non-pCR group. pCR did not significantly impact recurrence-free survival (RFS) or overall survival (OS). Multivariate analysis revealed that pretreatment N stage (clinical nodal status) was an independent prognostic factor for RFS and OS.

**Conclusions:**

*BRCA1* pathogenic variants, high Ki67 score and young age were predictors of pathological complete response, while clinical nodal status predicted survival outcomes in triple-negative breast cancer.

## Background

Breast cancer is the most common tumour in women in Latvia and worldwide; moreover, triple-negative breast cancer (TNBC) is diagnosed in 10–15% of all breast cancers and is characterized by rapid growth and shorter survival [1]. In comparison with other molecular subtypes of breast cancer, TNBC is more common in younger women [1]. Chemotherapy is currently the only treatment option for TNBC in Latvia. Anthracycline/taxane-based chemotherapy remains the standard of care systemic therapy for early-stage TNBC. TNBC is more sensitive to chemotherapy than other molecular subtypes, and 30–60% of patients can achieve a pathological complete response (pCR) to neoadjuvant chemotherapy (NAC), which is strongly associated with prolonged survival [2, 3]. Platinum-based NAC has been shown to increase the rates of pCR in TNBC patients compared to standard NAC; however, this treatment regimen has a higher level of toxicity [4].

Hereditary germline *BRCA* mutations occur in approximately 10–20% of women with stage I–III TNBC and play an important role in carcinogenesis and in predicting response to chemotherapy in TNBC with a characteristic pattern of DNA gains and losses [5]. Neoadjuvant platinum-based chemotherapy regimens increase the pCR rate by more than 60% in *BRCA1*-mutated breast cancer [6].

*The aim of this study* was to evaluate clinical and pathological factors that are associated with pCR in TNBC after neoadjuvant chemotherapy.

## Patients and methods

### Study population

Of the 130 TNBC patients treated between January 2018 and January 2020 in the Clinic of Oncology, Pauls Stradins Clinical University Hospital (Riga, Latvia), a total of 80 received NAC. Twenty-eight patients with metastatic TNBC and 26 patients with upfront surgery were excluded from the analysis. All patients were female. The clinical and pathological data of patients were collected from medical records and retrospectively analysed. Ethical approval was provided by the Ethics Committee at Latvian University, and the study was performed in accordance with ethical standards.

The clinical TNM stage was evaluated prior to NAC (cTNM) and after surgery (ypTNM). The tumour size and nodal status were evaluated by magnetic resonance imaging (MRI), and distant metastases were detected with CT scans.

Negative oestrogen receptors (ER), progesterone receptors (PgR), Ki67 and HER2 were diagnosed by core needle biopsy prior to NAC. The expression of ER, PrR and Ki67 was evaluated using immunohistochemistry (ICH) scoring of the percentage of cells with positive nuclear staining (1–100%). ER and PR were considered negative if nuclear staining was < 1%. Ki67 expression was considered low when < 50% and high when ≥ 50% stained cells were detected. HER2 was scored as 0 to 3 + by IHC. HER2 positivity was defined by an IHC score of 3 + or by HER2 gene amplification from FISH.

The response to NAC was evaluated by a pathologist following surgery. Pathological response to NAC was evaluated by the Miller-Payne grading system from I (no response to NAC) to V (complete pathological response). In our study, pCR was defined as the absence of any residual invasive cancer cells (ypT0N0M0).

Genomic DNA was isolated from peripheral blood cells and screened for the most common *BRCA*1 and *BRCA*2 pathogenic variants in Latvia (*BRCA*1: c.68_68del, c.181 T > G, c.4035delA, c.5266dupC, c.1961del, c.3700–3704, c.3756–3759, c.5117G > A, c.4675G > A, c.843_846del; *BRCA*2: c.643G > T/A, c.646del, c.658_659del, c.5946del, c1813dupA). Types of pathogenic variants are listed in Table 1.

**Table 1:** Description of study population (*n* = 80)

Characteristics	Total
**Total, n**	80
**Age, years**	
**Mean**	51.3
**Range**	26–80
**95% CI**	48.6–54
**Age group**	
**20–39**	17 (21.3)
**40–59**	38 (47.5)
** ≥ 60**	25 (31.2)
**Primary tumour diameter, mm**	
**Mean**	32.67
**Range**	10–70
**95% CI**	29.51–35.84
**T prior to NAC, n (%)**	
**cT1**	10 (12.5)
**cT2**	52 (65)
**cT3**	12 (15)
**cT4**	6 (7.5)
**N prior to NAC, n (%)**	
**cN0**	39 (48.8)
**cN1**	26 (32.5)
**cN2**	6 (7.5)
**cN3**	9 (11.2)
**T after surgery, n (%)**	
**ypT0**	27 (33.7)
**ypT1**	28 (35)
**ypT2**	19 (23.7)
**ypT3**	5 (6.3)
**ypT4**	1 (1.3)
**N after surgery, n (%)**	
**ypN0**	58 (72.5)
**ypN1**	17 (21.2)
**ypN2**	3 (3.8)
**ypN3**	2 (2.5)
**NAC regimen, n (%)**	
**Platinum containing**	47 (58.7)
**Nonplatinum containing**	33 (41.3)
**Germline *BRCA*1/2, n (%)**	
**BRCA1 pathogenic variant**	25 (31.2)
*BRCA1 c.5117G* > *A*	*6*
*BRCA1 c.5266dupC*	*11*
*BRCA1 c.4035delA*	*5*
*BRCA1 c.4675G* > *A*	*1*
*BRCA1 c.843_846del*	*1*
*BRCA1 c.181 T* > *G*	*1*
**BRCA2 pathogenic variant**	1 (1.3)
*BRCA2 c.1813dupA*	*1*
**BRCA1/2 wild type or unknown**	54 (67.5)
**Ki67, absolute count**	
**Mean**	47.3
**Range**	5–90
**95% CI**	42.6–52.0
**Ki67, n (%)**	
**Low (< 50%)**	40 (50)
**High (≥ 50%)**	40 (50)
**Pathological response (Miller-Payne), n (%)**	
**I-II**	19 (23.8)
**III-IV**	35 (43.7)
**V (pCR)**	26 (32.5)
**Surgery, n (%)**	
**Sectoral resection + sentinel node biopsy**	35 (43.8)
**Sectoral resection + lymph node excision**	6 (7.5)
**Mastectomy + sentinel node biopsy**	14 (17.5)
**Mastectomy + lymph node excision**	11 (13.7)
**Bilateral mastectomy**	14 (17.5)
**Recurrence, n (%)**	
**Present**	15 (18.7)
**Absent**	65 (81.3)
**Death, n (%)**	
**Dead**	9 (11.2)
**Alive**	71 (88.8)

*pCR* Pathological complete response, *NAC* Neoadjuvant chemotherapy

### Treatment

Patients were treated with platinum-containing and anthracycline- and taxane-based regimens. Regimens are listed in Table 2. The choice of chemotherapy regimen was made by a medical oncologist.

**Table 2:** NAC regimens of study patients

Regimen	All (*n* = 80), n	pCR (*n* = 26), n (% of all)
**Platinum noncontaining regimens**	**33**	**7 (21.2)**
*AC/EC*	2	1
*AC/EC plus paclitaxel*	19	5
*AC/EC plus docetaxel*	12	1
**Platinum containing regimens**	**47**	**19 (40.4)**
*Carboplatin* + *doxorubicin/epirubicin*	6	1
*Carboplatin* + *docetaxel*	11	6
*Carboplatin* + *docetaxel* + *AC/EC*	14	7
*Carboplatin* + *paclitaxel* + *AC/EC*	15	5
*Cisplatin* + *doxorubicin*	1	0

*AC* Doxorubicin, cyclophosphamide, *EC* Epirubicin, cyclophosphamide, *pCR* Pathological complete response

### Survival

Recurrence-free survival (RFS) was defined as the time from surgery to detection of local relapse or metastatic disease or death attributed to disease progression. Overall survival (OS) was defined as the time from surgery to death.

### Statistics

Associations between pCR, *BRCA* and clinicopathological characteristics were assessed with a Mann‒Whitney U test, Fisher’s exact test or Chi-square test. Kaplan‒Meier and log-rank tests were used to calculate survival differences. All analyses were performed using MedCalc statistical software, version 16.4.8 (MedCalc Software, Ostend, Belgium). *P* < 0.05 was considered to indicate statistical significance.

## Results

Eighty patients with TNBC received neoadjuvant chemotherapy at Pauls Stradins Clinical University Hospital (Riga, Latvia) between January 2018 and January 2020. Twenty-six patients (32.5%) had *BRCA1/2* pathogenic variants.

Twenty-six patients (32.5%) (*n* = 80) achieved pCR after NAC.

A total of 57.7% of *BRCA*-mutated patients and 40.4% of patients who received platinum-based NAC achieved pCR (Table 2).

### Pathological complete response

There was a statistically significant correlation between pCR and *BRCA1* pathogenic variants, high Ki67 levels and young age. pCR was detected in 57.7% *vs.* 20.4% (*p* = 0.001) in BRCA mutated *vs.* BRCA wild type; 47.5% *vs.* 17.5% (*p* = 0.007) in High Ki67 *vs.* low Ki67; 58.8% *vs.* 25.4% (*p* = 0.02) in age 20–39 *vs*. ≥ 40, respectively. The pCR rate was 40.4% *vs.* 21.2% (*p* = NS) in platinum-based *vs.* conventional NAC; 50% *vs.* 16.7% (*p* = NS) in T1 *vs.* T4; and 38.5% *vs.* 26.8% (*p* = NS) in N0 *vs.* N + , respectively (Table 3).

**Table 3:** Description of patients who achieved pCR

Characteristics	Total	pCR	Non-pCR	*p*
**Total, n**	80	26	54	
**Age, years**				0.08
**Mean**	51.3	46	53	
**Range**	26–80	32–69	26–80	
**95% CI**	48.6–54	36.7–61	47–58	
**Age group**				**0.02**
**20–39**	17 (21.3)	10 (58.8)	7(41.2)	
**40–59**	38 (47.5)	8 (21.1)	30 (78.1)	
** ≥ 60**	25 (31.2)	8 (32)	17 (68)	
**T prior to NAC, n (%)**				0.49
**cT1**	10 (12.5)	5 (50)	5 (50)	
**cT2**	52 (65)	17 (32.7)	35 (61.3)	
**cT3**	12 (15)	3 (25)	9 (75)	
**cT4**	6 (7.5)	1 (16.7)	5 (83.3)	
**N prior to NAC, n (%)**				0.63
**cN0**	39 (48.8)	15 (38.5)	24 (61.5)	
**cN1**	26 (32.5)	6 (23.1)	20 (76.9)	
**cN2**	6 (7.5)	2 (33.3)	4 (66.7)	
**cN3**	9 (11.2)	3 (33.3)	6 (66.7)	
**Primary tumour diameter, mm**				0.54
**Mean**	32.6	33.3	31.2	
**Range**	10–70	10–70	12–55	
**95% CI**	29.5–35.8	29.3–37.8	25.9–36.5	
**NAC regimen, n (%)**				0.09
**Platinum containing**	47 (58.7)	19 (40.4)	28 (59.6)	
**Nonplatinum containing**	33 (41.3)	7 (21.2)	26 (78.8)	
**Germline *BRCA*1/2, n (%)**				**0.001**
**Pathogenic variant**	26 (32.5)	15 (57.7)	11 (42.3)	
*BRCA1 c.5117G* > *A*	6 (7.5)	5 (83.3)	1 (16.7)	
*BRCA1 c.5266dupC*	11 (13.8)	7 (63.6)	4 (36.4)	
*BRCA1 c.4035delA*	5 (6.2)	1 (20)	4 (80)	
*BRCA1 c.4675G* > *A*	1 (1.25)	0 (0)	1 (100)	
*BRCA1 c.843_846del*	1 (1.25)	1 (100)	0 (0)	
*BRCA1 c.181 T* > *G*	1 (1.25)	1 (100)	0 (0)	
*BRCA2 c.1813dupA*	1 (1.25)	0 (0)	1 (100)	
**Wild type or unknown**	54 (67.5)	11 (20.4)	43 (79.6)	
**Ki67, n (%)**				**0.007**
**Low (< 50%)**	40 (50)	7 (17.5)	33 (82.5)	
**High (≥ 50%)**	40 (50)	19 (47.5)	21 (52.5)	

*pCR* Complete pathological response, *NAC* Neoadjuvant chemotherapy

### Survival

During the follow-up period (median 33 months, 95% CI 26–38 months), 15 patients progressed, and 9 died. In patients with recurrence, the mRFS was 15 months (10–41 months, 95% CI 11.6–25.3 months).

The mRFS in the study group (*n* = 80) was not met. The 1-y RFS was 95%, 2-y RFS was 84.2%, 3-y RFS was 80.5%, and 4-y RFS was 77.4%. mOS was also not met. The 1-y-OS was 100%, 2-y-OS was 96.1%, 3-y-OS was 88.7%, and 4-y-OS was 85.3%.

**Fig. 1:**
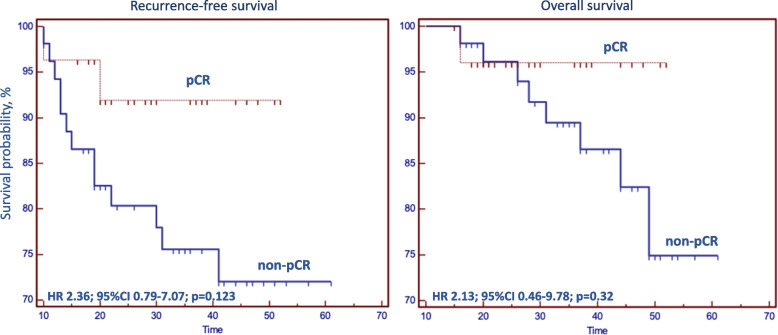
Kaplan‒Meier curves for the pathological complete response (pCR) on recurrence-free survival (RFS) and overall survival (OS)

RFS and OS differences in the pCR *vs.* non-pCR groups were not statistically significant. The hazard ratio for recurrence-free survival was 2.36 (95% CI 0.79–7.07; *p* = 0.123), and the HR for overall survival (OS) was 2.13 (95% CI 0.46–9.78; *p* = 0.32) (Fig. 1).

RFS was correlated with cT stage (mRFS in cT4 was 25 months; in other groups, mRFS was not met, *p* = 0.0067), cN stage (mRFS in cN2-3 was 31 months; in other groups, mRFS was not met, *p* = 0.0015), ypT stage (*p* = 0.0067), and ypN stage (*p* = 0.023).cN and ypN stage were correlated with longer OS – mOS in cN2-3 was 49 months *vs.* not met in cN0 (*p* = 0.007), but ypN0 *vs*. ypN + demonstrated HR 0.12 (95% CI 0.03–0.51; *p* = 0.003).cT, cN, ypT, and ypN stage were independent factors for RFS in univariate analysis, but cN stage was independent factor in multivariate analysis. cT, cN, ypT, ypN stage and platinum-based NAC were independent factors for OS in univariate analysis, but cN stage was independent factor in multivariate analysis (Table 4).

**Table 4:** Univariate and multivariate Cox proportional hazards regression for recurrence-free and overall survival

Covariate	**Univariate analysis**				**Multivariate analysis**		
	HR	95% CI	*p*		HR	95% CI	*p*
*Recurrence-free survival*							
cT	2.46	1.39–4.33	**0.0033**		1.43	0.70–2.89	0.3620
cN	2.89	1.49–5.60	**0.0016**		2.25	1.03–4.92	**0.0423**
ypT	2.14	1.33–3.43	**0.0025**		1.68	0.91–3.09	0.0940
ypN	1.95	1.20–3.18	**0.0171**		0.77	0.37–1.59	0.4880
BRCA	0.79	0.25–2.49	0.6952				
pCR	0.34	0.07–1.45	0.0971				
Platinum-based NAC	1.15	0.41–3.24	0.7852				
Ki67 group	0.51	0.17–1.48	0.2056				
Age group	1.49	0.71–3.13	0.2803				
*Overall survival*							
cT	2.20	1.09–4.44	**0.0274**	0.73		0.25–2.14	0.5698
cN	2.02	1.17–3.48	**0.0123**	3.42		1.00–11.62	**0.0502**
ypT	1.86	1.05–3.32	**0.0349**	1.27		0.53–3.05	0.5919
ypN	2.16	1.21–3.84	**0.0091**	0.90		0.40–2.81	0.4996
BRCA	0.77	0.16–3.72	0.7487				
pCR	0.36	0.04–2.94	0.3484				
Platinum-based NAC	7.02	0.88–55.62	**0.0213**	7.52		0.75–70.04	0.0885
Ki67 group	0.58	0.14–2.30	0.4283				
Age group	1.20	0.47–3.08	0.6995				

### Hereditary breast cancer

In the current cohort, 26 patients with germline *BRCA*1 or *BRCA*2 gene pathogenic variants were identified. Patients with *BRCA* pathogenic variants were younger (mean 44 years *vs*. 55 years, *p* = 0.017) and presented a better response to NAC (pCR 55.6% *vs.* 20.9%, *p* = 0.0003). In this group, platinum-based NAC was more frequently used (92.3% *vs.* 40.9%, *p* = 0.0002), and mastectomy was the preferred surgery option (*p* = 0.0001). Statistically significant differences in CA15-3, CA125, platelet count, Ki67 score, primary tumour size, clinical TNM stage and recurrence were not observed (Table 5).

**Table 5:** Clinical characteristics in *BRCA*-mutated and *BRCA*-negative groups. Patients with unknown *BRCA* status were excluded from the current analysis

Characteristic	*BRCA-*mutated	*BRCA-negative*	*p*
Total, n	26	49	
*BRCA*1	25	NA	
*BRCA*2	1	NA	
Age, years			**0.017**
Range	33–80	26–71	
Mean	44	55	
95% CI	36.8–48.5	48–60	
Ki67 score, %			0.621
Range	5–87	10–90	
Mean	60	52.5	
95% CI	40–60	39.5–60	
Pathological response (Miller-Payne), n (%)			**0.0139**
I-II	0 (0)	17 (34.7)	
III-IV	11 (42.3)	22 (44.9)	
V (pCR)	15 (57.7)	10 (20.4)	
Size, mm			0.929
Range	10–63	12–70	
Mean	32.5	29	
95% CI	20.1–40.4	25–36.3	
T prior to NAC, n (%)			0.141
cT1	4 (15.4)	6 (12.2)	
cT2	18 (69.2)	29 (59.2)	
cT3	4 (15.4)	8 (16.3)	
cT4	0 (0)	6 (12.3)	
N prior to NAC, n (%)			0.589
cN0	12 (46.2)	25 (51)	
cN1	9 (34.6)	16 (32.6)	
cN2	2 (7.7)	4 (8.2)	
cN3	3 (11.5)	4 (8.2)	
NAC regimen, n (%)			**0.0001**
Platinum based	24 (92.3)	20 (40.8)	
Nonplatinum based	2 (7.7)	29 (59.2)	
Surgery, n (%)			**0.0001**
Sectoral resection + sentinel node biopsy	5 (19.2)	27 (55.1)	
Sectoral resection + lymph node excision	0 (0)	5 (10.2)	
Mastectomy + sentinel node biopsy	4 (15.4)	9 (18.4)	
Mastectomy + lymph node excision	4 (15.4)	7 (14.3)	
Bilateral mastectomy	13 (50)	1 (2)	

*NAC* Neoadjuvant chemotherapy, *pCR* Pathological complete response

## Discussion

Our findings from the retrospective study at Pauls Stradins Clinical University Hospital suggested that *BRCA* pathogenic variants and high Ki67 expression are associated with a higher incidence of complete pathological response after neoadjuvant chemotherapy.

As one of the prognostic biomarkers in the treatment of breast cancer, the Ki-67 index has been demonstrated to be associated with tumour chemosensitivity and associated with a more frequent pCR, while pCR improves patient survival [2]. In a study by Nakashoji, a high Ki67 score was observed in 83% of patients in the pCR group *vs.* 46% in the non-pCR group [7]. In this study, 50% of patients receiving neoadjuvant chemotherapy had a Ki-67 index above 50%, and 47.5% of these patients achieved a pCR. Additionally, pCR was observed more frequently in patients with *BRCA* 1 or 2 pathogenic variants (57.7%).

The addition of platinum to NAC regimens showed promising results, but their use remains controversial. The meta-analysis performed by Li and his colleagues shows that the addition of platinum to standard chemotherapy increases the probability of pCR by 13.2% (49.1% in the platinum-based NAC group *vs.* 35.9% in the standard NAC group) [8], but in a study published in 2018 by Gass and colleagues, pCR reached 50% after platinum/taxane treatment (*vs.* 41.8% after anthracycline/taxane treatment) [9]. Similarly, in the GeparSixto trial, carboplatin-based NACT increased pCR rates – 53.2% *vs*. 36.9% (*p* = 0.005) [4], while our analysis failed to support these findings in this retrospective cohort – 40.4% in platinum-based NAC *vs*. 21.2% in nonplatinum-based NAC, but this was not statistically significant in the adjusted analysis.

In patients with TNBC, a pCR has been observed to be a strong indicator for better outcome. If pCR is achieved as a result of NAC, survival is similar to survival in other, more favourable, molecular subtypes of BC, but in the case of a partial response to NAC, short survival and fast recurrence are commonly observed [9]. In a study by Gass and colleagues, pCR was significantly related to increased RFS and OS [10]. Two other randomized trials, the CALB40603 trial and BrighTNess study, demonstrated significant increases in pCR rates and relapse-free survival in TNBC with the addition of carboplatin to taxane- and anthracycline-containing NACT [11, 12]. The BrighTNess study identified significant improvements in RFS for patients with pCR *vs*. non-pCR both in patients with an identified germline pathogenic variant in *BRCA*1 or *BRCA*2 (HR 0.14) and in *BRCA* wildtype patients (HR 0.29) [12]. Our study confirmed these data – patients with pCR experienced increased RFS (7.7% relapsed in pCR *vs*. 24.1% in non-pCR) and OS (3.8% died in pCR *vs.* 14.8% in non-pCR) than patients with partial response, but the result was not statistically significant, which may be based on the low number of patients in the study group.

Regarding decreased survival rates, patients with incomplete response to NAC are candidates for postoperative systemic treatment, such as chemotherapy or innovative drugs, to improve disease control and survival. Since 2017, when Masud and colleagues published results from the CREATE-X study with colleagues, it is known that patients with residual disease following NAC and surgery may benefit from adjuvant chemotherapy with capecitabine [13]. Capecitabine was not reimbursed in Latvia in the current time period, and only 4 patients received adjuvant treatment in the non-pCR group.

In our study, a comparison between TNBC patients with *BRCA* pathogenic variants and sporadic cancer patients was also performed. As expected, patients with *BRCA* pathogenic variants were significantly younger, and increased pathological response to NAC was observed. According to the Miller-Payne grading system, grade I-II response (no response or weak response to NAC) was not observed (0%) in the *BRCA*-mutated group compared to 34.7% in the sporadic TNBC group, but pCR was observed in 57.7% *vs.* 20.4%, respectively. Patients with *BRCA*-mutated TNBC were more often treated with platinum-based NAC, and the mastectomy rate was significantly higher. Similar findings were published by Kedzierawski and colleagues – the rate of pCR in *BRCA*-mutated TNBC was 54.2% *vs*. 40.3% [14].

This study has limitations that should be mentioned. First, it was a retrospective study with a relative deficiency of patients in subgroups, which could have influenced the bias of the obtained results. The collected sample size in two years was small, which could be improved in further studies by adding patients from the next years. Second, the study group was heterogeneous – different chemotherapy regimens used, clinical and pathological findings, surgery types, and *BRCA* status (mutated, wild type or unknown) could also impact the results. In our study, ten different platinum-based chemotherapy regimens with different counts of chemotherapy agents were used. In such a small study, it is difficult to refer to the results, but we observed a benefit of adding platinum salts in patients with BRCA1/2 pathogenic variants. Larger randomized trials have already been published addressing the efficacy of platinum-based NAC and its correlation with pCR and survival in TNBC; therefore, our retrospective study adds real-life experience from a single university hospital to the current knowledge despite its limitations. Further studies are needed to confirm the current results.

## Conclusions

This retrospective study observed higher pCR rates after neoadjuvant therapy in TNBC patients with younger age, higher Ki67 score and *BRCA1* pathogenic variants. Additionally, *BRCA* pathogenic variants were associated with young age, increased response to NAC, higher rate of pCR, increased mastectomy rate and frequent use of platinum-based neoadjuvant chemotherapy.

This study did not confirm the impact of platinum-based NAC on survival in TNBC due to the small patient number and heterogeneous list of chemotherapy regimens used.

## Data Availability

The datasets generated during and/or analysed during the current study are available from the corresponding author on reasonable request.

## Abbreviations

BC: Breast cancer
ER: Oestrogen receptor
NAC: Neoadjuvant chemotherapy
OS: Overall survival
pCR: Complete pathological response
PrR: Progesterone receptor
RFS: Recurrence-free survival
TNBC: Triple-negative breast cancer
